# Two-year clinical performance of dual- and light-cure bulk-fill resin composites in Class ӀӀ restorations: a randomized clinical trial

**DOI:** 10.1007/s00784-024-05538-0

**Published:** 2024-02-07

**Authors:** Mohamed Elshirbeny Elawsya, Marmar Ahmed Montaser, Noha Abdel-Mawla El-Wassefy, Nadia Mohamed Zaghloul

**Affiliations:** 1 Department of Conservative Dentistry, Faculty of Dentistry, Mansoura University, Algomhoria Street, P.O. Box 35516, Mansoura, Aldakhlia Egypt; 2 Department of Dental Biomaterials, Faculty of Dentistry, Mansoura University, Mansoura, Egypt; 3 Department of Dental Biomaterials, Faculty of Dentistry, Mansoura National University, Mansoura, Egypt

**Keywords:** Bulk-fill resin composite, Dual-cure, Light-cure, Class ӀӀ restorations, Clinical performance, Randomized clinical trial

## Abstract

**Objective:**

This study aimed to compare the clinical performance of dual- and light-cure bulk-fill resin composites (BFRCs) in Class ӀӀ restorations after 2 years.

**Materials and methods:**

A double-blinded, prospective, randomized clinical trial (RCT) was conducted following the CONSORT (Consolidated Standard of Reporting Trials) guidelines. Forty patients were enrolled in the study. Each patient received three compound Class ӀӀ restorations. One dual-cure (Fill-Up; Coltene Waledent AG) and two light-cure (QuiXfil; Dentsply, and Tetric N-Ceram Bulk Fill; Ivoclar Vivadent) BFRCs were used for 120 Class ӀӀ restorations. A universal adhesive (ONE COAT 7 UNIVERSAL; Coltene Waledent AG) was used with all restorations. Restorations were clinically evaluated after 1 week (baseline), 6 months, 12 months, 18 months, and finally after 24 months using the FDI World Dental Federation (FDI) criteria. The Kruskal–Wallis test was used for comparison between BFRCs groups at baseline and at each recall period, and the Wilcoxon signed-rank test was used for comparing different follow-up times of each BFRC to baseline. The level of significance was set at *p* < 0.05.

**Results:**

All BFRCs restorations showed only minor changes and revealed no statistically significant differences between their clinical performance for all evaluated parameters at all recall periods; also, there was no statistically significant difference between all recall periods and baseline for all evaluated parameters.

**Conclusion:**

The two-year clinical performance of dual-cure BFRC was comparable to light-cure BFRCs in Class ӀӀ restorations.

**Clinical relevance:**

Dual- and light-cure BFRCs showed excellent clinical performance in Class ӀӀ restorations after a 2-year clinical follow-up.

## Introduction

A new dimension has been given to esthetic and conservative dentistry by resin-based composites (RBCs) as restorative dental materials, by improving their clinical handling, mechanical properties, and ability to mimic natural teeth appearance [1]. However, many factors limit RBCs performance, especially depth of cure and degree of conversion [2, 3]. Despite great advances in RBCs technologies, insufficient depth of cure and polymerization shrinkage are considered two of its major disadvantages [4–6]. Polymerization shrinkage of RBCs ranges from 2 to 6% of volumetric shrinkage and is considered one of the most important causes of RBCs failure by generating stresses at tooth/restoration interface and jeopardizing the bonding integrity over time. Furthermore, polymerization stresses induce leakage at cavity margins leading to marginal staining and development of carious lesions [7, 8].

When restoring cavities with light-cure RBCs, the incremental technique with a maximum 2 mm layer thickness has been considered the gold standard for placement and curing resin composite in layers of limited thickness [7]. However, the incremental technique and light curing each layer separately is a time-consuming procedure for both the operator and patient. The incremental technique also increases the possibility of moisture contamination or air bubbles inclusion between the individual layers of RBCs restorations [9].

With advancement in polymer chemistry, curing lights, and photo-activation technologies, a new type of resin composites, called bulk-fill resin composites (BFRCs), has emerged that can be applied in 4–5 mm layer thickness without adverse effects on polymerization shrinkage, cavity adaptation, or degree of conversion [9]. Moreover, the manufacturers stated that the polymerization shrinkage of BFRCs is even less than that of conventional RBCs. Consequently, polymerization shrinkage problems can be reduced [10]. The placement of larger resin composite increments may reduce the time needed for posterior restorations placing and thereby reduce technique sensitivity.

The improvement in depth of cure of light-cure BFRCs is usually obtained by an increased translucency of the resin composite, more content of photo-initiator, or an additional photo-initiator type [11]. Even with these improvements, light-cure BFRCs can still suffer from insufficient deep layers polymerization because of attenuation or impeded access of the curing light [12]. Therefore, when restoring Class II cavities that have deep margins, it is common practice to apply additional photo-polymerization from both the buccal and lingual sides of the tooth after removing the matrix band [13, 14]. This is done to account for the greater distance between the tip of the light curing device and the resin composite layer near the gingival margin.

Another type of resin composites has dual curing polymerization reactions [15]. These dual-cure resin composites have both light curing and chemical curing polymerization systems. The light curing polymerization reaction achieves fast initial hardening of the resin composite top layer enabling finishing and polishing procedures. While the resin composite deep layers that receive insufficient curing light are polymerized by the slower chemical-cure polymerization reaction. Therefore, dual-cure BFRCs can provide a higher degree of conversion and unlimited depth of cure due to the effective and depth-independent chemical-cure polymerization system [9, 16, 17]. In addition to this clinical advantage over light-cure resin composite, dual-cure resin composite polymerizes more slowly, decreasing the polymerization shrinkage stresses as a result [18]. However, dual-cure resin composites exhibit a lower color stability due to aromatic tertiary amines in their formulation that produce a yellowing effect on the resin material in the long term [19, 20].

Laboratory evaluations are important for the early assessment of the dental restorative materials, but only clinical studies can take into account all of the potential variables (which differ from patient to patient) that influence the overall performance of the dental restorative materials [21–24]. These variables include humidity variations, temperature fluctuations, mastication forces, abrasive foods, chemically active fluids and foods, salivary enzymes, and bacterial byproducts [25–27].

Although several clinical studies have investigated the performance of light-cure BFRCs [28–31], there is no any published clinical study on dual-cure BFRCs until present, according to the knowledge of the authors. Hence, further investigation in this particular area is needed. The null hypothesis tested in this study was that there would be no difference in the 2-year clinical performance of all tested BFRCs in Class II restorations.

## Materials and methods

### Materials

Three BFRCs were used, including one dual-cure BFRC (Fill-Up; Coltene Waledent AG, Altstatten, Switzerland) and two light-cure BFRCs (QuiXfil; Dentsply, Konstanz, Germany, and Tetric N-Ceram Bulk Fill; Ivoclar Vivadent, AG, Schaan, Liechtenstein). All materials used in the current study and their descriptions are presented in Table 1.

**Table 1:** Materials used in the study

	Manufacturer	Lot no	Shade	Type	Composition	Filler load wt%/vol%
Fill-Up	Coltene Waledent AG, Altstatten, Switzerland	J38436	Universal	Medium-viscosity micro-hybrid dual-cure bulk-fill	TMPTMA, UDMA, TEGDMA, Bis-GMA, dibenzoyl peroxide, benzoyl peroxide, zinc oxide coated	65/49
QuiXfil	Dentsply, Konstanz, Germany	0504	Universal	High-viscosity sculptable micro-hybrid light-cure bulk-fill	UDMA, TEGDMA, di and tri-methacrylate, strontium-alumino-sodium-fluoro-phosphate-silicate glass	86/66
Tetric N-Ceram Bulk Fill	Ivoclar Vivadent, AG, Schaan, Liechtenstein	Y48204	IVA	High-viscosity sculptable nano-hybrid light-cure bulk-fill	Bis-GMA, Bis-EMA, UDMA, barium glass, ytterbium trifluoride, mixed oxide, and copolymers	80/60
N-Etch Etching Gel	Ivoclar Vivadent, AG, Schaan, Liechtenstein	X27261	N/A	Etching gel	37% phosphoric acid	N/A
ONE COAT 7 UNIVERSAL	Coltene Waledent AG, Altstatten, Switzerland	J32343	N/A	Universal adhesive	HEMA, hydroxypropyl-methacrylate, MMA-modified polyacrylic acid, UDMA, amorphous silicic, 10-MDP	N/A
One Coat 7.0 Activator	Coltene Waledent AG, Altstatten, Switzerland	J67279	N/A	Activator (for chemical- and dual-cure materials)	Ethanol, water, activator	N/A

Abbreviations: *Bis-GMA*, bisphenol A-diglycidyl dimethacrylate; *Bis-EMA*, bisphenol-A ethoxylated dimethacrylate; *UDMA*, urethane dimethacrylate; *TMPTMA*, trimethylolpropane trimethacrylate; *TEGDMA*, triethylene glycol dimethacrylate

### Ethical considerations

This study was submitted to and approved by the Dental Research Ethics Committee (Faculty of Dentistry, Mansoura University) under protocol number A01150620 and registered in ClinicalTrials.gov PRS (https://register.clinicaltrials.gov) under identification number NCT06137989. According to the guidelines of Mansoura University institution’s ethics committee, each participant signed a written informed consent for participation in this study. Participants were free to leave the study at any time. The study was conducted following the CONSORT (Consolidated Standard of Reporting Trials) guidelines [32].

### Sample size calculation

Assuming a power of 80% and a significance level of 0.05, the sample size was calculated using G*Power 3.1 (Heinrich Heine University, Düsseldorf, Germany) based on the results of a similar study design conducted by Guney et al. [33]. According to this calculation, the minimum sample size required per group was 33. With the potential dropouts in consideration, the trial included 40 patients because three restorations (one of each group) were made in each patient.

### Inclusion and exclusion criteria

Patients were chosen at random from the Mansoura University Faculty of Dentistry's outpatient clinic (23 males and 17 females). Patients’ selection achieved a balance in age from 21 to 50 with an average age of 35 years old. The participation of patients in the follow-up was requested. Inclusion criteria were that each patient should have at least three permanent molars and premolars that need to be treated with small to medium-sized compound Class II restorations due to primary carious lesions (ICDAS 4, or 5). All patients are required to have a full and normal occlusion and to maintain adequate oral hygiene. General criteria for exclusion were heavy bruxism, poor oral hygiene, chronic or severe periodontitis, and a history of allergies to any of the materials utilized in this study (pregnant or nursing females were excluded). Specific exclusion criteria were fractured or visibly cracked teeth, rampant caries, faulty restoration opposite or adjacent to the tooth to be restored, and atypical extrinsic staining.

### Study design

**Fig. 1:**
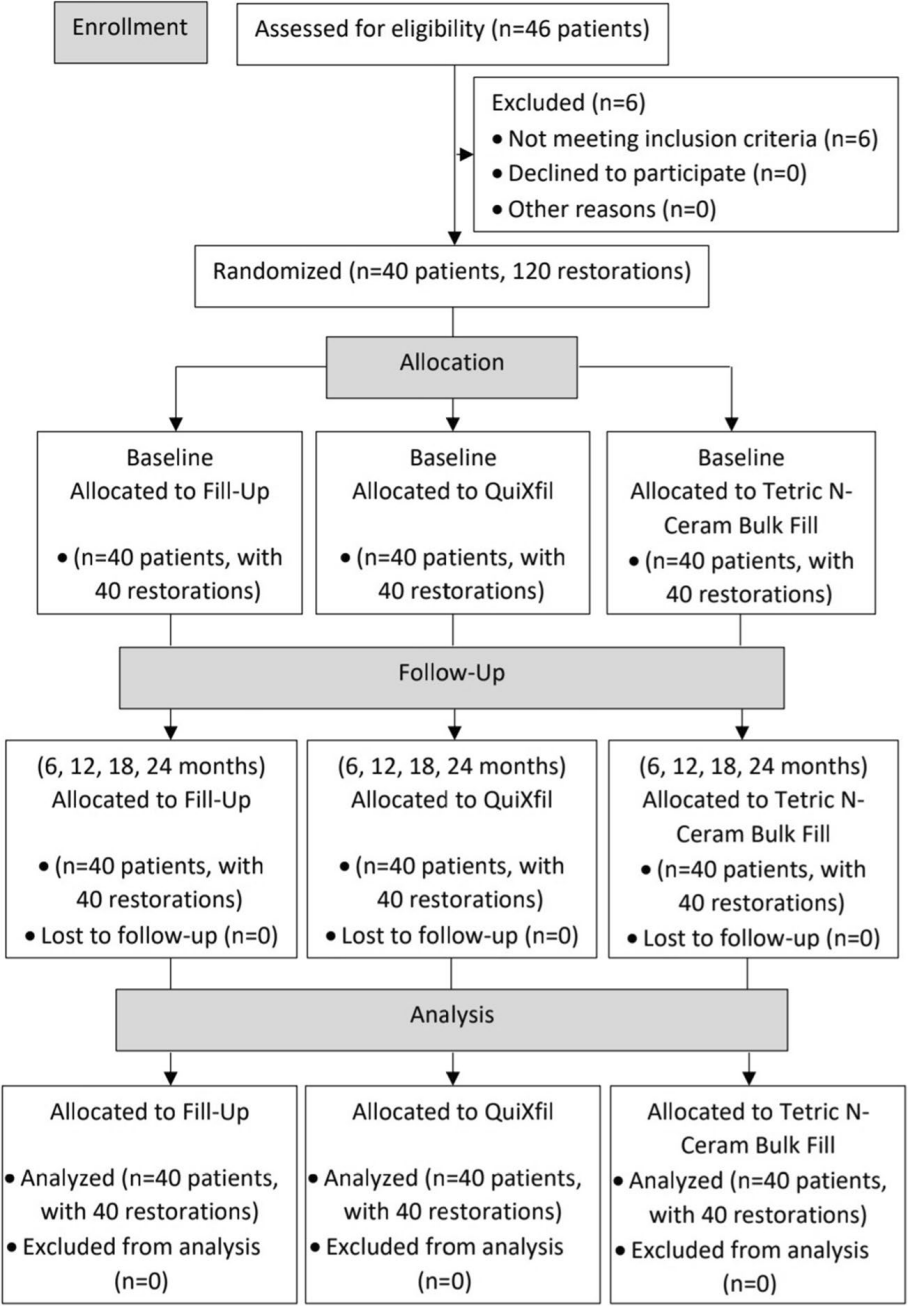
The flow chart of the study

A double-blinded prospective randomized clinical trial (RCT) was designed to evaluate and compare the clinical performance of three BFRCs. The flow chart of the study is displayed in Fig. 1. To be included in the current study, each patient should have three primary carious lesions to receive three compound Class ӀӀ restorations. A total of 120 compound Class ӀӀ restorations were placed in 82 molars and 38 premolars. Each patient received three compound Class ӀӀ restorations with the three tested BFRCs, in order to make intra-individual comparison possible. For randomization of the three restorations in each patient, sealed envelopes were used for both tooth number and restoration type [34]. Except the operator, the patients and the two clinical examiners were blinded to the type of BFRC applied in each tooth.

### Clinical procedures

Periapical and bitewing radiographs were taken for the teeth to be restored prior to restorative procedures; also, vitality test scores were obtained using a pulp vitality tester (Kerr Vitality Scanner; Kerr, Peterborough, UK).

#### Cavity preparation

**Fig. 2:**
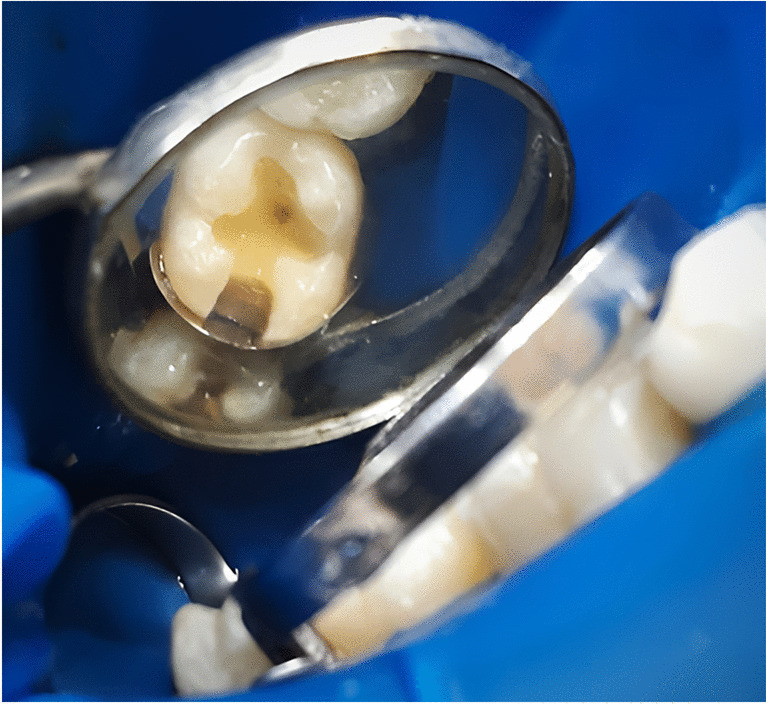
Representative image of compound Class II (occluso-distal) cavity preparation in tooth #47

Local anesthesia (Artinibsa 4% 1:100.000, Inibsa Dental S.L.U, Spain) was given at the beginning to prevent any discomfort for the patient during the restorative procedures. Round diamond points (S6801.FG.012, Komet, Brasseler, Lemgo, Germany) for enamel and flat end straight fissure carbide burs # 56 (H21-009-FG, Komet, Brasseler, Lemgo, Germany) for dentine at high-speed hand piece (Sirona T3, Bensheim, Germany) with water-cooling system were used for cavities preparation. Slow-speed tungsten carbide burs (Excavabur RA ISO 012, Dentsply LH, LTD, UK) and hand excavators (#52, Dentsply, Maillefer, Switzerland) were utilized for deep caries excavation. The preparations were limited to that required for caries eradication, and excessive removal of sound tooth structure was avoided as much as possible. Assessment of the excavated preparation floor was performed using conventional tactile and visual methods, and the excavation was terminated when the dentine was hard on probing. The final dimensions of all cavities were determined with a periodontal probe with depth (distance between the cavo-surface margin and the pulpal floor) ranged between 3 and 4 mm. The following were the features of the preparation design: (1) no cusps involvement in the cavity preparations; (2) above the gingival sulcus, sound enamel was present on all of the gingival margins; (3) the margins and walls of the preparation were not beveled; and (4) the preparations’ bucco-lingual width did not extend beyond one-third of the inter-cuspal distance (Fig. 2).

#### Restorative procedures

All restorative procedures were performed under rubber dam (Dentsply LH, LTD, UK) isolation. Only in two preparations, pulp shadow was observed—without pulp exposure—after caries removal. Calcium hydroxide-based material (Dycal, Dentsply, Caulk, Milford, DE, USA) was placed over this area in the cavity, then sealed by resin-modified glass ionomer liner ( GC Fuji lining, GC, America). For restoring all Class II cavity preparations, a sectional metal matrix system (TOR VM, Russia) was used. This system consists of a round ring and a special-designed band to reestablish the proximal contact area anatomy. A proper-sized wedge (TOR VM, Russia) was inserted for adequate band adaptation at the gingival area. A full water rinse was used to thoroughly clean the cavities.

BFRCs used in the study were Fill-Up (Coltene Waledent AG, Altstatten, Switzerland), QuiXfil (Dentsply, Konstanz, Germany), and Tetric N-Ceram Bulk Fill (Ivoclar Vivadent, AG, Schaan, Liechtenstein). For all restorations, 37% phosphoric acid gel: N-Etch Etching Gel (Ivoclar Vivadent, AG, Schaan, Liechtenstein) was used for selective etching of all enamel margins for 15 s followed by thorough water rinsing and gentle air-drying. A universal adhesive: ONE COAT 7 UNIVERSAL (Coltene Waledent AG, Altstatten, Switzerland) was used with all restorations. For QuiXfil and Tetric N-Ceram Bulk Fill restorations (light-cure BFRCs), two layers of adhesive were applied using an applicator brush to the entire cavity preparation with 20 s rubbing of the first layer, followed by 5 s gentle air-drying, then light-cured for 10 s. For Fill-Up restorations (dual-cure BFRC), one layer of adhesive was applied with 20 s rubbing, followed by 5 s gentle air-drying. Then, one drop of the adhesive was mixed with one drop of an activator for chemical- and dual-cure materials: One Coat 7.0 Activator (Coltene Waledent AG, Altstatten, Switzerland) and applied, followed by 5 s gentle air-drying and finally light-cured for 10 s.

For QuiXfil and Tetric N-Ceram Bulk Fill restorations, resin composite material was placed with a thickness not more than 4 mm, adapted by a resin composite instrument (Optra Contact, Optra Sculpt, Ivoclar Vivadent), and light-cured for 20 s. Also, after removing the matrix band, the proximal areas were light-cured additionally from the buccal and lingual embrasures for 10 s each. For Fill-Up restorations, the resin composite material was injected directly into the cavity from a dual-chamber syringe with an auto-mix tip. The resin composite material was injected from the deepest area of the cavity until complete cavity filling in one bulk increment and then light-cured for 10 s. Light curing of adhesive and BFRCs restorations was achieved according to the manufacturer’s instructions by an LED curing light (Elipar Deep Cure; 3 M ESPE, St. Paul, MN, USA) with 1200 mW/cm^2^ irradiance. The output power of the light curing unit was verified regularly by a light radiometer (Bluephase Meter II, Ivoclar Vivadent, Liechtenstein).

Articulating papers (Bausch, Nashua, NH, USA) were used to check premature occlusal contacts after removing the rubber dam. Occlusal prematurities were removed, and restoration finishing was done by water-cooled, fine-grit yellow-coded flame diamond stones (#368EF-021 Extra Fine Bud FG, Komet, USA). Polishing was performed by rubber points and polishing brushes (Occlubrush, Kerr, Switzerland) with a low-speed contra-angle handpiece (NAC-EC, NSK, Japan) under water coolant and minimal pressure with a maximum speed of 20,000 rpm.

#### Evaluation procedures

Prior to evaluating the study cases, the two clinical examiners participated in two training sessions, each consisting of ten similar clinical cases. The intra-class correlation coefficient and Cohen’s kappa coefficient were used to evaluate the intra- and inter-examiner agreements. More than 90% intra- and inter-examiner agreement was necessary for the calibration of evaluations. All BFRCs restorations were clinically evaluated after 1 week (baseline), 6 months, 12 months, 18 months, and finally after 24 months by two independent clinical examiners (not allowed to be involved in the restorative procedures) using the FDI World Dental Federation (FDI) criteria [35]. At all recall intervals, clinical intraoral photographs were taken, and throughout the evaluation processes, scores of FDI criteria were recorded using a standardized case report for each patient.

The following parameters that were relevant to this study were selected to be evaluated: surface luster, staining (surface, margin), color match and translucency, esthetic anatomical form, fracture of material and retention, marginal adaptation, occlusal wear, proximal contact, radiographic examination, postoperative (hyper-) sensitivity and tooth vitality, recurrence of caries, and tooth integrity (enamel cracks, tooth fractures). Each criterion is exhibited by five scores (1, 2, 3, 4, 5), three scores for acceptable (1, 2, 3) and two scores for non-acceptable (4 for reparable and 5 for replacement). The detailed description of FDI criteria and scoring system is shown in Table 2.

**Table 2:** FDI criteria and scores

A. Esthetic properties	1. Surface luster	2. Staining a. surface b. margin	3. Color match and translucency	4. Esthetic anatomical form	B. Functional properties	5. Fracture of material and retention	6. Marginal adaptation	7. Occlusal wear	8. Proximal contact	9. Radiographic examination	C. Biological properties	10. Postoperative (hyper-)sensitivity and tooth vitality	11. Recurrence of caries	12. Tooth integrity (enamel cracks, tooth fractures)
1. Clinically excellent/very good	1.1 Luster comparable to enamel	2a.1 No surface staining 2b.1 No marginal staining	3.1 Good color match, no difference in shade and/or translucency	4.1 Form is ideal	1. Clinically excellent/very good	5.1 No fractures/cracks	6.1 Harmonious outline, no gaps, no white or discolored lines	7.1 Wear corresponding to 80–120% of enamel	8.1 Normal contact point (floss can pass)	9.1 No pathology, harmonious transition between restoration and tooth	1. Clinically very good	10.1 No hypersensitivity, normal vitality	11.1 No secondary or primary caries	12.1 Complete integrity
2. Clinically good (after polishing probably very good)	1.2.1 Slightly dull, not noticeable from speaking distance 1.2.2 Some isolated pores	2a.2 Minor surface staining, easily removable by polishing 2b.2 Minor marginal staining, easily removable by polishing	3.2 Minor deviations in shade and/or translucency	4.2 Form is only slightly deviated from the normal	2. Clinically good	5.2 Small hairline crack	6.2.1 Marginal gap (< 150 μm), white lines 6.2.2 Small marginal fracture removable by polishing 6.2.3 Slight ditching, slight step/flashes, minor irregularities	7.2 50–80% or 120–150% wear compared to that of corresponding enamel	8.2. Contact slightly too strong but no disadvantage (floss can only pass with pressure)	9.2.1 Acceptable cement excess present 9.2.2 Positive/negative step present at margin < 150 μm	2. Clinically good (after correction maybe very good) No treatment required	10.2 Minor hypersensitivity for a limited period of time, normal vitality	11.2 Small and localized demineralization	12.2.1 Small marginal enamel fracture (< 150 μm) 12.2.2 Hairline crack in enamel (< 150 μm)
3. Clinically sufficient/satisfactory (minor shortcomings, no unacceptable effects but not adjustable without damage to the tooth)	1.3.1 Dull surface but acceptable if covered with film of saliva 1.3.2 Multiple pores on more than one-third of the surface	2a.3 Moderate surface staining that may also present on other teeth, not esthetically unacceptable 2b.3 Moderate marginal staining, not esthetically unacceptable	3.3 Distinct deviation but acceptable. Does not affect esthetics: 3.3.1 more opaque 3.3.2 more translucent 3.3.3 darker 3.3.4 brighter	4.3 Form deviates from the normal but is esthetically acceptable	3. Clinically sufficient/satisfactory (minor shortcomings, no unacceptable effects but not adjustable without damage to the tooth)	5.3 Two or more or larger hairline cracks and/or material chip fracture not affecting the marginal integrity or proximal contact	6.3.1 Gap < 250 μm not removable 6.3.2. Several small marginal fractures 6.3.3 Major irregularities, ditching or flash, steps	7.3 < 50% or 150–300% of corresponding enamel	8.3. Somewhat weak contact, no indication of damage to tooth, gingiva or periodontal structures; 50 μm metal blade can pass	9. 3. 1 Marginal gap < 200 μm 9. 3. 2 Negative steps visible < 250 μm. no adverse effects noticed 9.3.3 Poor radiopacity of filling material	3.Clinically sufficient/satisfactory (minor shortcomings with no adverse effects but not adjustable without damage to the tooth)	10.3.1 Moderate hypersensitivity 10.3.2 Delayed/mild sensitivity; no subjective complaints, no treatment needed	11.3 Larger areas of demineralization (only preventive measures necessary)	12.3.1 Marginal enamel defect < 250 μm 12.3.2 Crack < 250 μm 12.3.3 Enamel chipping 12.3.4 Multiple cracks
4. Clinically unsatisfactory (but reparable)	1.4.1 Rough surface, cannot be masked by saliva film, simple polishing is not sufficient. Further intervention necessary 1.4.2 Voids	2a.4 Unacceptable surface staining on the restoration and major intervention necessary for improvement 2b.4 Pronounced marginal staining; major intervention necessary for improvement	3.4 Localized clinically deviation that can be corrected by repair: 3.4.1 Too opaque 3.4.2 Too translucent 3.4.3 Too dark 3.4.4 Too bright	4.4. Form is affected and unacceptable esthetically Intervention/correction is necessary	4. Clinically unsatisfactory/(but reparable)	5.4.1 Material chip fractures which damage marginal quality or proximal contacts 5.4.2 Bulk fractures with partial loss (less than half of the restoration)	6.4.1 Gap > 250 μm or dentine/base exposed 6.4.2. Severe ditching or marginal fractures 6.4.3 Larger irregularities or steps (repair necessary)	7.4 Restoration > 300% of enamel wear or antagonist > 300%	8.4 Too weak and possible damage due to food impaction; 100 μm metal blade can pass	9.4.1 Marginal gap > 250 μm 9.4.2 Cement excess accessible but not removable 9.4.3 Negative steps > 250 μm and repairable	4. Clinically unsatisfactory (repair for prophylactic reasons)	10.4.1 Intense hypersensitivity 10.4.2 Delayed with minor subjective symptoms 10.4.3 No clinical detectable sensitivity. Intervention necessary but not replacement	11. 4 Caries with cavitation and suspected undermining caries (localized and accessible can be repaired)	12.4.1 Major marginal enamel defects; gap > 250 μm or dentine or base exposed 12.4.2 Large cracks > 250 μm, probe penetrates 12.4.3. Large enamel chipping or wall fracture
5. Clinically poor (replacement necessary)	1.5 Very rough, unacceptable plaque retentive surface	2a.5 Severe surface staining and/or subsurface staining, generalized or localized, not accessible for intervention 2b.5 Deep marginal staining, not accessible for intervention	3.5 Unacceptable Replacement necessary	4.5 Form is unsatisfactory and/or lost. Repair not feasible/reasonable Replacement needed	5. Clinically poor (replacement necessary)	5.5 (Partial or complete) loss of restoration or multiple fractures	6.5.1 Restoration (complete or partial) is loose but in situ 6.5.2 Generalized major gaps or irregularities	7.5 Restoration or antagonist > 500% of corresponding enamel	8.5 Too weak and/or clear damage due to food impaction and/or pain/gingivitis	9.5.1 Secondary caries, large gaps 9.5.2 Apical pathology 9.5.3 Fracture/loss of restoration or tooth	5. Clinically poor (replacement necessary)	10.5 Intense, acute pulpitis or non-vital tooth. Endodontic treatment is necessary, and restoration has to be replaced	11.5 Deep caries or exposed dentine that is not accessible for repair of restoration	12.5. Cusp or tooth fracture

#### Evaluation methods

Patients were asked for teeth brushing before attending for each evaluation. A magnifying loupe (3.5 x) with a powerful light source was used to improve clinical visibility. Two special probes with different blunt tips (150 and 250 µm) and dental floss were used to evaluate marginal adaptation. Also, proximal contact was evaluated with dental floss. Periapical and bitewing radiographs were taken for radiographic examination. Postoperative sensitivity was assessed by blowing a stream of compressed air for 3 s at a 2–3 cm distance from the restoration. The vitality was tested using dry ice—CO_2_ snow—(Odontotest, Fricar A.G. Zurich, Switzerland). According to ICDAS, recurrent caries was diagnosed.

#### Occlusal wear evaluation

**Fig. 3:**
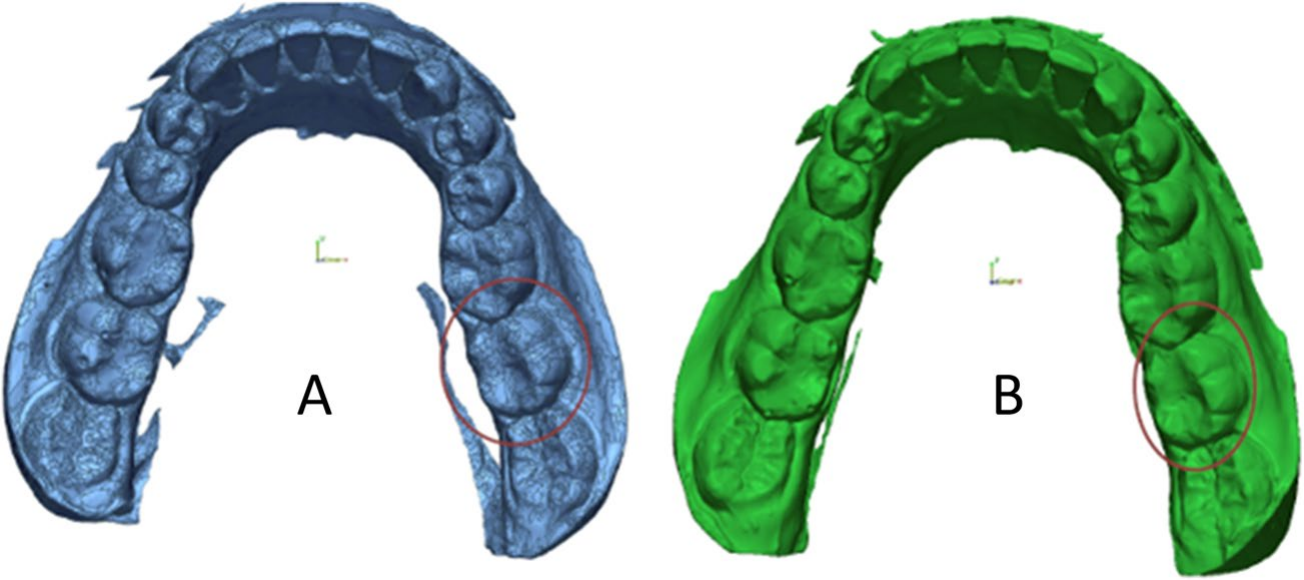
Scanned replicas (**A** Baseline replica, **B** 2-year follow-up replica)

**Fig. 4:**
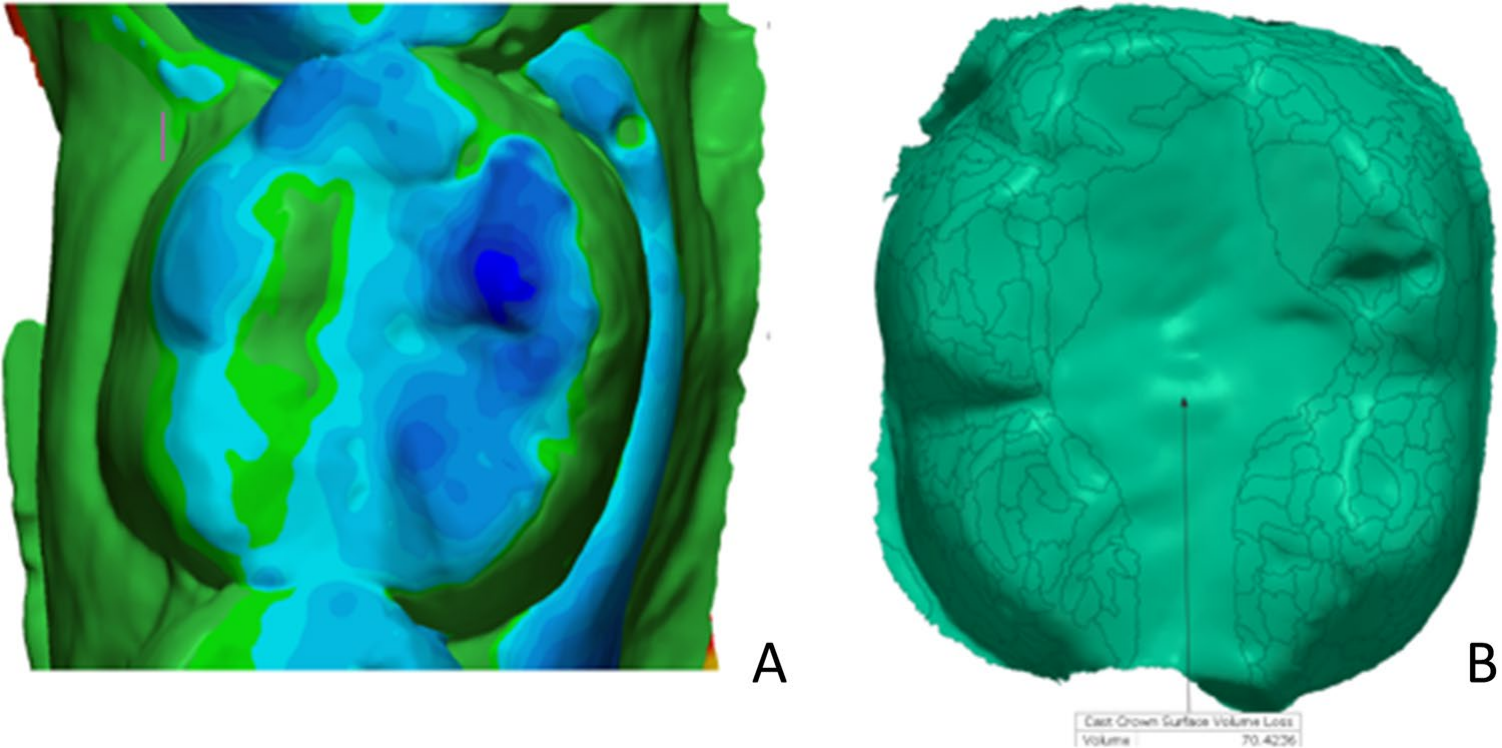
**A** Best fit alignment between baseline and 2-year follow-up replicas. **B** Quantification procedure of the amount of wear between baseline and 2-year follow-up

At baseline and each recall, an impression was taken with polyvinyl siloxane impression material. A stone gypsum GC Fujirock EP White (Dental stone type IV, GC Europe, Leuven, Belgium) was used for impression pouring. All replicas were uniformly trimmed. Using a 3D laser scanner (Medit T710, Medit Corp, Seoul, Korea), all gypsum replicas were scanned three-dimensionally (Fig. 3). A 3D analysis software (Geomagic Control, 3D Systems, NC, USA) was used to superimpose each follow-up image on the baseline image separately, using three user-defined references for best-fit alignment [36]. Each follow-up image was digitally subtracted from the baseline image by the software, and a differential image for each follow-up produced by this digital subtraction was obtained (Fig. 4). The entire occlusal surface was identified manually and represented the total occlusal volume loss. The restoration area was also identified manually and represented the restoration volume loss (restoration wear), and by subtraction of the restoration volume loss from the total occlusal volume loss, the result was the enamel volume loss (enamel wear) [37]. The wear of the restoration per unit area (mm^2^) was compared to the wear of the corresponding enamel per unit area (mm^2^), and the FDI wear score was given.

### Statistical analysis

Statistical analyses were carried out using SPSS 22 software (IBM SPSS Inc., Chicago, IL, USA). Number and percent described qualitative data. After exploring the data distribution (Shapiro–Wilk test), it showed a non-parametric distribution. The Kruskal–Wallis test was used for comparison between BFRCs groups at baseline and at each recall period, and the Wilcoxon signed-rank test was used for comparing different follow-up times of each BFRC to baseline. The level of significance was determined at *p* < 0.05.

## Results

At all recall periods, all patients attended and none of them reported negative appreciation for restorative procedures that were performed. All BFRCs restorations showed only minor changes, and score 1 was the majority of scores for all evaluated parameters. All BFRCs restorations revealed no statistically significant differences between their clinical performance for all evaluated parameters at all recall periods (*p* > 0.05), and also there was no statistically significant difference between all recall periods and baseline for all evaluated parameters (*p* > 0.05), as shown in Table 3.

**Table 3:** Clinical evaluation scores of all tested BFRCs restorations according to FDI criteria at baseline, 6-, 12-, 18-, and 24-month follow-up with results of Kruskal–Wallis and Wilcoxon signed-rank tests

Category		Score	Baseline (*n* = 40)			6-month (*n* = 40)			12-month (*n* = 40)			18-month (*n* = 40)			24-month (*n* = 40)			FU $	QF $	TNB $
			FU	QF	TNB	FU	QF	TNB	FU	QF	TNB	FU	QF	TNB	FU	QF	TNB			
Esthetic properties	Surface luster	1	40	40	40	40	40	40	39	40	40	38	38	40	37	38	40			
		2	-	-	-	-	-	-	1	-	-	2	2	-	3	2	-	p2 = 0.317		
		3	-	-	-	-	-	-	-	-	-	-	-	-	-	-	-	p3 = 0.157	p3 = 0.157	
		4	-	-	-	-	-	-	-	-	-	-	-	-	-	-	-	p4 = 0.083	p4 = 0.157	
		5	-	-	-	-	-	-	-	-	-	-	-	-	-	-	-			
	#								*p* = 0.368			*p* = 0.358			*p* = 0.235					
	Staining: (a)margin	1	40	40	40	40	40	40	40	40	40	40	40	38	38	39	38			
		2	-	-	-	-	-	-	-	-	-	-	-	2	2	1	2			
		3	-	-	-	-	-	-	-	-	-	-	-	-	-	-	-	p4 = 0.157	p4 = 0.317	p3 = 0.157
		4	-	-	-	-	-	-	-	-	-	-	-	-	-	-	-			p4 = 0.157
		5	-	-	-	-	-	-	-	-	-	-	-	-	-	-	-			
	#											*p* = 0.133			*p* = 0.813					
	Staining: (b)surface	1	40	40	40	40	40	40	40	40	40	40	40	40	40	40	40			
		2	-	-	-	-	-	-	-	-	-	-	-	-	-	-	-			
		3	-	-	-	-	-	-	-	-	-	-	-	-	-	-	-			
		4	-	-	-	-	-	-	-	-	-	-	-	-	-	-	-			
		5	-	-	-	-	-	-	-	-	-	-	-	-	-	-	-			
	#																			
	Color match and translucency	1	40	40	40	40	40	40	40	40	40	40	40	40	38	40	40			
		2	-	-	-	-	-	-	-	-	-	-	-	-	2	-	-			
		3	-	-	-	-	-	-	-	-	-	-	-	-	-	-	-	p4 = 0.157		
		4	-	-	-	-	-	-	-	-	-	-	-	-	-	-	-			
		5	-	-	-	-	-	-	-	-	-	-	-	-	-	-	-			
	#														*p* = 0.133					
	Esthetic anatomical form	1	40	40	40	40	40	40	40	40	40	40	40	40	40	40	40			
		2	-	-	-	-	-	-	-	-	-	-	-	-	-	-	-			
		3	-	-	-	-	-	-	-	-	-	-	-	-	-	-	-			
		4	-	-	-	-	-	-	-	-	-	-	-	-	-	-	-			
		5	-	-	-	-	-	-	-	-	-	-	-	-	-	-	-			
	#																			
Functional properties	Fracture of material and retention	1	40	40	40	40	40	40	40	40	40	40	40	40	40	40	40			
		2	-	-	-	-	-	-	-	-	-	-	-	-	-	-	-			
		3	-	-	-	-	-	-	-	-	-	-	-	-	-	-	-			
		4	-	-	-	-	-	-	-	-	-	-	-	-	-	-	-			
		5	-	-	-	-	-	-	-	-	-	-	-	-	-	-	-			
	#																			
	Marginal adaptation	1	40	40	40	40	40	40	40	40	40	40	39	40	39	37	38			
		2	-	-	-	-	-	-	-	-	-	-	1	-	1	3	2			
		3	-	-	-	-	-	-	-	-	-	-	-	-	-	-	-	p4 = 0.317	p3 = 0.317	p4 = 0.157
		4	-	-	-	-	-	-	-	-	-	-	-	-	-	-	-		p4 = 0.083	
		5	-	-	-	-	-	-	-	-	-	-	-	-	-	-	-			
	#											*p* = 0.368			*p* = 0.593					
	Occlusal wear	1	40	40	40	40	40	40	40	40	40	39	40	40	38	40	40			
		2	-	-	-	-	-	-	-	-	-	1	-	-	2	-	-			
		3	-	-	-	-	-	-	-	-	-	-	-	-	-	-	-	p3 = 0.317		
		4	-	-	-	-	-	-	-	-	-	-	-	-	-	-	-	p4 = 0.157		
		5	-	-	-	-	-	-	-	-	-	-	-	-	-	-	-			
	#											*p* = 0.368			*p* = 0.133					
	Proximal contact	1	40	40	40	40	40	40	40	40	40	40	40	40	40	40	40			
		2	-	-	-	-	-	-	-	-	-	-	-	-	-	-	-			
		3	-	-	-	-	-	-	-	-	-	-	-	-	-	-	-			
		4	-	-	-	-	-	-	-	-	-	-	-	-	-	-	-			
		5	-	-	-	-	-	-	-	-	-	-	-	-	-	-	-			
	#																			
	Radiographic examination	1	40	40	40	40	40	40	40	40	40	40	40	40	40	40	40			
		2	-	-	-	-	-	-	-	-	-	-	-	-	-	-	-			
		3	-	-	-	-	-	-	-	-	-	-	-	-	-	-	-			
		4	-	-	-	-	-	-	-	-	-	-	-	-	-	-	-			
		5	-	-	-	-	-	-	-	-	-	-	-	-	-	-	-			
	#																			
Biological properties	Post-operative sensitivity	1	38	39	37	40	40	40	40	40	40	40	40	40	40	40	40			
		2	2	1	3	-	-	-	-	-	-	-	-	-	-	-	-	p1 = 0.157	p1 = 0.317	p1 = 0.083
		3	-	-	-	-	-	-	-	-	-	-	-	-	-	-	-	p2 = 0.157	p2 = 0.317	p2 = 0.083
		4	-	-	-	-	-	-	-	-	-	-	-	-	-	-	-	p3 = 0.157	p3 = 0.317	p3 = 0.083
		5	-	-	-	-	-	-	-	-	-	-	-	-	-	-	-	p4 = 0.157	p4 = 0.317	p4 = 0.083
	#		*p* = 0.593																	
	Recurrent caries	1	40	40	40	40	40	40	40	40	40	40	40	40	40	40	39			
		2	-	-	-	-	-	-	-	-	-	-	-	-	-	-	1			
		3	-	-	-	-	-	-	-	-	-	-	-	-	-	-	-			p4 = 0.317
		4	-	-	-	-	-	-	-	-	-	-	-	-	-	-	-			
		5	-	-	-	-	-	-	-	-	-	-	-	-	-	-	-			
	#														*p* = 0.368					
	Tooth integrity	1	40	40	40	40	40	40	40	40	40	40	40	40	40	40	40			
		2	-	-	-	-	-	-	-	-	-	-	-	-	-	-	-			
		3	-	-	-	-	-	-	-	-	-	-	-	-	-	-	-			
		4	-	-	-	-	-	-	-	-	-	-	-	-	-	-	-			
		5	-	-	-	-	-	-	-	-	-	-	-	-	-	-	-			
	#																			

*FU*, Fill-Up; *QF*, QuiXfil; *TNB*, Tetric N-Ceram Bulk Fill

^#^USED test: Kruskal–Wallis test for comparing between BFRCs groups (FU, QF&TNB)

^$^USED test: Wilcoxon signed-rank test for comparing different follow-up times to baseline (p1, comparison between baseline and 6-month; p2, comparison between baseline and 12-month; p3, comparison between baseline and 18-month; p4, comparison between baseline and 24-month)

### Esthetic properties

Regarding surface luster criterion, score 2 was recorded at 12-month recall in one Fill-Up restoration and at 18-month recall in two Fill-Up and two QuiXfil restorations. At 24-month recall, three Fill-Up and two QuiXfil restorations recorded score 2 for surface luster.

Regarding the marginal staining criterion, two scores 2 were recorded at 18-month recall in two Tetric N-Ceram Bulk Fill restorations. At 24-month recall, two Fill-Up, one QuiXfil, and two Tetric N-Ceram Bulk Fill restorations recorded score 2.

Regarding color match and translucency, only two Fill-Up restorations recorded score 2 at 24-month recall.

Regarding surface staining and esthetic anatomical form, all tested BFRCs restorations recorded score 1 at baseline and at all recall visits.

### Functional properties

At 6- and 12-month follow-ups, there were no marginal defects recorded at the margins for all restorations. At 18-month follow-up, one QuiXfil restoration recorded score 2 for marginal adaptation. At 24-month follow-up, one Fill-Up, three QuiXfil, and two Tetric N-Ceram Bulk Fill restorations were rated score 2 for marginal adaptation.

Concerning occlusal wear, score 2 was recorded at 18-month recall in one Fill-Up restoration and at 24-month recall in two Fill-Up restorations.

Regarding proximal contact, radiographic examination, and fracture of material and retention, all tested BFRCs restorations recorded score 1 at baseline and at all recall visits.

### Biological properties

Regarding the post-operative sensitivity criterion, two Fill-Up restorations, one QuiXfil restoration, and three Tetric N-Ceram Bulk Fill restorations reported score 2 post-operative sensitivity at baseline. At 6-month, 12-month, 18-month, and 24-month follow-up periods, all tested BFRCs restorations recorded score 1 for post-operative sensitivity. Restorations that received calcium hydroxide then sealed by resin-modified glass ionomer liner recorded score 1 for post-operative sensitivity at baseline and at all follow-up periods.

Concerning recurrence of caries, only one case reported score 2 recurrent caries for Tetric N-Ceram Bulk Fill restoration at a 24-month follow-up period.

Regarding tooth integrity, all tested BFRCs restorations recorded score 1 at all recall visits.

## Discussion

One of the major achievements of contemporary biomaterials research is resin composite restorative materials, which replace biological tissue in both function and appearance [38]. Modern dentistry is now based on resin composite materials and adhesive techniques. Despite advancements in technology, polymerization shrinkage of resin composites still poses a problem and places restrictions on their usage clinically [39]. This polymerization shrinkage affects the adhesive layer holding the restorative material to the tooth, frequently leading to bond failure and marginal infiltration [40]. These issues have motivated manufacturers to provide us with alternatives and develop products that are quicker and easier to use, both in terms of the material and the technique. As a result, BFRCs materials have been developed with the purpose of time and thus cost savings [9].

While laboratory testing may be valuable for learning about a filling material’s prospective performance and handling, they are insufficient for assessing a material’s clinical performance or handling characteristics. Concerns about the clinical durability of these tooth-colored restorations cannot be resolved by in vitro investigations only. Reproduction of oral physiology is challenging due to the complexity of various intraoral environmental condition variables, including occlusal stress, temperature fluctuations, bacterial flora, and pH changing. Therefore, while evaluating dental materials or restoration techniques, only the clinical environment may be relevant [41]. Although several studies have investigated the clinical performance of light-cure BFRCs, no studies up till now have investigated the clinical performance of dual-cure BFRCs. Therefore, this study investigated the clinical performance of one dual-cure (Fill-Up) in comparison with two light-cure (QuiXfil and Tetric N-Ceram Bulk Fill) BFRCs.

Many factors affect the longevity and durability of dental restorations. Materials and techniques utilized, patient compliance with oral hygiene, and patient susceptibility to caries are some of these. The majority of the patients included in this study had good oral hygiene and no periodontal disorders along the time of evaluation. In order to make intra-individual comparison possible, each patient in this study received three compound Class ӀӀ restorations with the three tested BFRCs.

The adhesive bonding efficiency and adhesive strategy have a significant role in the increased longevity of resin composite restorations [42, 43]. In this study, a universal adhesive was used with a selective enamel etching technique for all restorations. Because it has been reported that the universal adhesives showed highly good clinical performance when used with selective etching of the enamel margins of the cavity [42].

For clinical trials that evaluate the effectiveness of resin-based composite restorations, objective, relevant, and reliable criteria are required. In the current study, BFRCs Class II restorations were evaluated using FDI criteria that were defined by Hickel et al. [44] and were approved by the Science Committee of the FDI World Dental Federation in 2007 and were considered in 2008 as “Standard Criteria.” Therefore, their use was indicated in clinical studies evaluating dental restorations regarding their materials, application techniques, and interventions, as well as in clinical practice to decide whether a restoration should be maintained, repaired, or replaced. The FDI criteria were described as being practical (diverse and easily accessible criteria), relevant (sensitive as well as suitable for current restorative materials and clinical study design), and standardized (making comparisons between different investigations easier) [45]. According to the FDI criteria, wear scores can be obtained qualitatively by the clinical examiner or quantitatively on replicas with a 3D scanner and computer software. In the current study, we used the quantitative method for more accuracy.

Only one skilled operator placed all of the restorations in the current study since the factors that influence the clinical result depend more on the operator than the material evaluated [46]. This made sure that all restorations were carried out consistently and under the same circumstances. It is recommended to utilize resin-based composites in small to medium-sized cavities rather than extensive restorations to reduce direct occlusal contacts, despite the fact that they have been widely used to restore posterior teeth. On the basis of this approach, small to medium-sized cavities were considered for the clinical cases. The current study favored butt joint, clean-cut, non-beveled preparations in the occlusal cavities to a beveled cavo-surface design. A thin margin of restorative material produced by a beveled preparation may fracture and leave a ledge-type defect in the marginal regions. To avoid salivary contamination in this study, all restorations were completed under rubber dam isolation. In this study, calcium hydroxide was applied and sealed by resin-modified glass ionomer liner in two preparations in which pulp shadow was observed, as cytotoxicity of monomers could not be ignored in this situation. Previous studies concluded that calcium hydroxide does not affect post-operative sensitivity [47, 48].

Regarding the surface luster, there were no significant differences found between all BFRCs restorations along the follow-up period. However, Fill-Up and QuiXfil recorded score 2 for surface luster, while Tetric N-Ceram Bulk Fill did not. This might be due to the different filler sizes between Fill-Up, QuiXfil (micro-hybrid fillers), and Tetric N-Ceram Bulk Fill (nano-hybrid fillers), as smaller size filler could retain surface polishing better [49].

Previous study has shown that the three tested BFRCs (Fill-Up, QuiXfil, and Tetric N-Ceram Bulk Fill) provided adequate degree of conversion, microhardness, and depth of cure [5]. In the current study, the good results of marginal staining, marginal adaptation, color match, and recurrent caries might be attributed to the low polymerization shrinkage, adequate degree of conversion, and depth of cure of the tested BFRCs. Also, the excellent results of anatomical form, proximal contact, radiographic examination, and wear resistance in this study might be attributed to the good mechanical properties and microhardness of the tested BFRCs.

The post-operative sensitivity is related to many factors as the procedure of cavity preparation, adhesive approach, leakage, occlusal discrepancies, cuspal deformation by shrinkage stress, type of resin composite, and placement technique of the resin composite [50, 51]. Regarding post-operative sensitivity in the current study, all the tested BFRCs were clinically accepted. This might be attributed to the low polymerization shrinkage of the tested BFRCs, small to medium-sized cavity preparations, using universal adhesive with selective etching technique. Afifi et al. [50] conducted a randomized clinical study with similar findings.

After 2 years of clinical service, all evaluated BFRCs restorations were classified as acceptable and recorded either score 1 or score 2 for all the evaluated parameters. Score 1 was the most common score for the majority of the restorations. Previous clinical studies have also shown similar results confirming the good clinical performance of BFRCs materials for posterior teeth restorations [28, 30, 31, 33]. The null hypothesis was accepted as, following the clinical follow-up period, there were no significant differences observed between all BFRCs restorations for all evaluated parameters.

However, the high success rate presented in the current study may have resulted from the restorations being completed in the optimum possible conditions and being performed on teeth that satisfied the previously defined inclusion and exclusion criteria. One of the limitations of this clinical investigation is that 24 months may be a short period for substantial changes to become noticeable regarding the clinical performance of the three BFRCs in Class II restorations. Thus, further studies should evaluate their long-term clinical performance.

## Conclusions

Based on the results of this clinical study, it can be concluded that.

1. The 2-year clinical performance of dual-cure BFRC was comparable to that of light-cure BFRCs in compound Class ӀӀ restorations.
2. The three tested BFRCs showed excellent clinical performance in compound Class ӀӀ restorations after a 2-year clinical follow-up.

## Data Availability

The data that support the findings of this study are available from the corresponding author upon reasonable request.
